# Assessment of Initial Oxygenation Levels of Chronic Obstructive Pulmonary Disease (COPD) and Their Impact on Basis and Vital Tools: Retrospective Cohort Study From India

**DOI:** 10.7759/cureus.75470

**Published:** 2024-12-10

**Authors:** Sonali Ghosh, Ram K Brahmachari, Susmita Ghosh, Sourav Das Choudhury, Kaushik Ghosh

**Affiliations:** 1 Emergency Medicine and Critical Care, Institute of Post-Graduate Medical Education and Research and Seth Sukhlal Karnani Memorial Hospital, Kolkata, IND; 2 Medicine, Murshidabad Medical College and Hospital, Berhampore, IND; 3 Anaesthesiology, Murshidabad Medical College and Hospital, Berhampore, IND; 4 Internal Medicine, Nibedita Healthcare, Berhampore, IND; 5 Medicine, KPC Medical College and Hospital, Kolkata, IND

**Keywords:** copd: chronic obstructive pulmonary disease, copd patients, decaf indices, news2 indices, oxygenation levels

## Abstract

Introduction

Chronic obstructive pulmonary disease (COPD) is a significant contributor to global morbidity and mortality. Despite well-established management protocols, treatment remains suboptimal due to high costs and mortality rates. This study aims to compare the impact of initial oxygenation status, Dyspnea, Eosinopenia, Consolidation, Acidemia, and Atrial Fibrillation (DECAF), and National Early Warning Score 2 (NEWS2) scores on management outcomes in COPD patients.

Methods

In this retrospective study, we analyzed 100 consecutive patients admitted for COPD exacerbation. Patients were categorized into four groups based on admission oxygen saturation (SpO2): Group A (≤87%), Group B (88-92%), Group C (93-96%), and Group D (97-100%). Data collected included oxygen saturation, chest X-rays, laboratory findings, DECAF, and NEWS2 scores.

Results

The mean age of the cohort was 68.54 ± 10.95 years. Groups A and B (SpO2 ≤ 93-96%) had significantly higher rates of hypercapnia (50%), non-invasive ventilation use (63%), and prolonged hospital stays (15%) compared to Groups C and D (p < 0.05). A strong correlation was found between initial SpO2 and both DECAF (p = 0.04) and NEWS2 (p = 0.001) scores. DECAF correlated with arterial oxygen (pO2) and carbon dioxide (pCO2) levels, while NEWS2 was linked with pCO2, albumin, and white blood cell (WBC) counts (p < 0.05). Both DECAF and NEWS2 predicted longer hospital stays (p < 0.05).

Conclusion

An initial SpO2 ≤ 93-96% was an independent predictor of higher hypercapnia rates, extended hospitalization, and increased use of non-invasive ventilation. This emphasizes the importance of initial oxygenation status in the clinical assessment of COPD patients.

## Introduction

Chronic obstructive pulmonary disease (COPD) is distinguished by enduring respiratory symptoms and restricted airflow resulting from irregularities in the airway and/or suboptimal alveolar function, typically triggered by substantial exposure to harmful particles or gases [1]. According to a report published in 2019, COPD was ranked as the fourth leading cause of death, particularly in India [2]. In India, the prevalence of COPD is alarmingly high, with studies reporting rates between 1.6% and 26.6% among non-smokers [3]. Despite being highly prevalent, COPD is underdiagnosed, especially at primary healthcare levels, partly due to the underuse of spirometry. Spirometry measures the volume and flow of air during inhalation and exhalation; hence, it is paramount in diagnosing COPD [4]. It is underused in primary care, thus delaying diagnosis and resulting in increased mortality. Conventional spirometric indices, however, are relatively insensitive for the detection of early-stage COPD and for differential diagnosis from other pulmonary diseases [5]. It is common for these indices to miss even the presence of extensive small airway disease - a hallmark of COPD - thereby requiring more sensitive diagnostic techniques [6].

With COPD, pulmonary function declines, and the likelihood of alveolar hypoxia and hypoxemia rises with disease progression, signaling the advanced disease stage [7-10]. Hypoxemia in COPD patients causes a decreased quality of life, reduced skeletal muscle function, and, ultimately, increased risk of death [11,12]. Long-term oxygen therapy is the ideal therapeutic intervention that has been shown to prolong life in COPD patients with hypoxemia [13-16]. Monitoring oxygen saturation (SpO2) levels, typically using a non-invasive oximeter, is a standard practice in assessing the severity of hypoxemia and guiding oxygen therapy. However, while SpO2 measurement is crucial, its role in conjunction with other prognostic tools, such as the Dyspnea, Eosinopenia, Consolidation, Acidemia, and Atrial Fibrillation (DECAF) and National Early Warning Score 2 (NEWS2) scores, has not been fully explored in COPD management [17,18].

In 2012, Steer et al. [19] developed a comprehensive score called the DECAF score, which aims to forecast the likelihood of death within a hospital setting in COPD. DECAF is a simple tool that can be administered at the bedside using indices routinely available on admission. DECAF scores of zero to one are strong predictors of survival, and DECAF scores of four to six are strong predictors of mortality [20,21]. It is imperative to constantly monitor patients who have been identified as high-risk and implement prompt medical measures in order to effectively mitigate the entire mortality rate [20]. The DECAF score also improves hospital facility utilization by reducing inappropriate admissions and helping flag high-risk patients needing emergency interventions [22].

The NEWS2, an updated version of NEWS developed in the UK, is increasingly used globally because it uses readily available parameters and is well-validated [23-25]. The parameters included are (1) respiration rate, (2) oxygen saturation, (3) systolic blood pressure (SBP), (4) pulse rate, (5) level of consciousness, and (6) body temperature. This revised version allows for adjustments to be made in patients with both low and inappropriately high oxygen saturations and is, therefore, particularly relevant for conditions such as COPD [26,27]. NEWS2 has been shown to decrease mortality risk and identify a patient's requirement for non-invasive ventilation (NIV) and prolonged hospitalization, which may be of use in the management of COPD exacerbations [28,29].

Despite the proven efficacy of the DECAF and NEWS2 scores in various settings, their combined use with initial SpO2 levels in managing COPD has not been thoroughly investigated, particularly in the Indian context. India bears a significant burden of respiratory diseases, with COPD contributing to approximately 20% of global cases annually and a mortality rate of 8.7% [30]. The high cost of COPD management and the underutilization of established diagnostic protocols contribute to elevated in-hospital mortality rates and extended hospital stays [31,32]. While some studies from India have demonstrated the utility of the DECAF score in identifying high-risk patients, the NEWS2 score has been primarily applied during the COVID-19 pandemic and has not been widely used in COPD management [33-35].

This retrospective study aims to assess the impact of initial oxygenation (SpO2) status, DECAF score, and NEWS2 score on the management of COPD. By evaluating these parameters together, this study seeks to provide insights that could lead to improved clinical decision-making and better outcomes for COPD patients in India. The findings may encourage the adoption of these prognostic tools in routine clinical practice, ultimately enhancing the quality of care for COPD patients.

## Materials and methods

Participants

A retrospective observational study was conducted at Murshidabad Medical College and Hospital, West Bengal, India. The patients aged ≥ 18 years who were admitted to the hospital for the exacerbation of COPD between 2023 and 2024 were included in the study. The sample size was calculated (taking the area under the receiver operating characteristic (AUROC) curve for in-hospital mortality of 0.83 with 95% CI and 7.36% margin of error in consideration). A total of 100 patients met these criteria. Patients who were either receiving non-invasive domiciliary ventilation or having existing comorbidities that limit survival to less than one year or with multiorgan failure or with coexisting fibrotic lung disease, pulmonary thromboembolism, and significant cardiovascular disease that influence survival were excluded. Finally, 100 consecutive patients who fulfilled the inclusion and exclusion criteria were included in the study.

The hospital records of these patients, including demographics, smoking history, and vitals such as peripheral capillary oxygen saturation (SpO2), pulse and respiratory rate, blood pressure, and temperature, were recorded on admission. The SpO2 levels were obtained from all patients by an oximeter while breathing room air. The patients whose pCO2 exceeded 45 mmHg were categorized as having hypercapnia following standard clinical guidelines. The responsiveness of the patients was assessed using an AVPU scale, where patients were categorized as follows: Alert (A), responsive to Voice (V), responsive to Pain (P), or Unresponsive (U). They also included the blood gas levels on admission (pH, pO2, pCO2, HCO3), blood investigations, total and differential leucocyte count, C-reactive protein (CRP), and albumin. All the patients had undergone X-ray chest examinations. The COPD-associated comorbidities, such as diabetes, cor pulmonale, cerebrovascular disease, ischemic heart disease (IHD), and left-sided heart failure, were documented. Left ventricular failure was diagnosed typically with transthoracic echo of reduced ejection fraction of 45% or less. Cor pulmonale was diagnosed with clinical signs of right-sided heart failure or radiological evidence of right-sided volume overload. The DECAF and NEWS2 scores were also calculated. Written permission was obtained from the head of the department of medicine to access the data from the coronary care unit (CCU), patient confidentiality was strictly maintained throughout the study, and the data were anonymized. Based on their oxygen saturation (SpO2) at the time of admission, patients were classified into the following four groups (Table 1).

**Table 1:** Oxygen saturation (SpO2)

Groups	Group A	Group B	Group C	Group D
Oxygen saturation level	87% or less	88-92%,	93-96%	97-100%

Statistical analysis

All statistical analyses were carried out using IBM SPSS Statistics for Windows, Version 28.0.1.1 (IBM Corp., Armonk, NY). Hypothesis testing was conducted at the 5% (two-sided) significance level. A probability value of <0.05 was considered as significant. Categorical variables will be reported as numbers (percentages). Two proportion z-tests (one-tailed) were used to compare the groups. The continuous data were represented with mean ± SDd and median (range). The comparison of continuous variables among categorical variables was done using either the ANOVA or Kruskal-Wallis test based on the distribution of the data. The association between categorical variables was done using the chi-square test. Karl Pearson's correlation coefficient was calculated to justify the relationship between continuous values.

## Results

**Figure 1:**
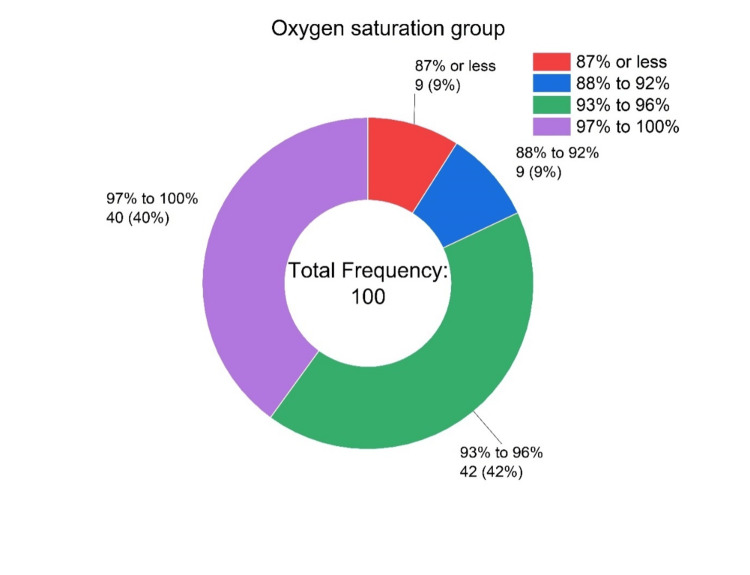
The number and percentage of patients in the four oxygen saturation groups

Table 2 shows the baseline characteristics of the 100 patients. The mean age of the four SpO2 groups was around 68 years, with more males (87%) than females (13%). The median pulse (beats/minute) and respiratory rates showed an initial increase from Group A (pulse rate: 101 beats/minute; respiratory rate: 23 breaths/minute) to Group B (pulse rate: 102 beats/minute; respiratory rate: 28 breaths/minute) and then gradually fell in Group D (pulse rate: 98 beats/minute; respiratory rate: 22 breaths/minute). The SBP, diastolic blood pressure (DBP), and body temperature across the four groups were well within the normal range. Moreover, of the 100 patients, only 18 belonged to the first two groups (Figure 1).

**Table 2:** Demographic and baseline characteristics of the patients with COPD (analyzed using ANOVA and chi-square test) The continuous data have been presented in terms of mean ± SD and median(range). The categorical data have been presented in frequency(%). p < 0.05 is considered a significance level.

Variables		Oxygen saturation level groups						
		Total (n = 100)	Group A (≤87%) (n = 9)	Group B (88- 92%) (n = 9)	Group C (93-96%) (n = 42)	Group D (97-100%) (n = 40)	F/chi square	p-value
Age (years)	Mean ± SD	68.54±10.95	69.3±9.39	67.8±5.76	69.6±11.76	66.3±9.68	0.531	0.573
	Median (range)	69 (30-92)	68 (48-88)	69 (55-81)	70 (30-90)	68 (47-92)		
Gender	Male	87 (87%)	9 (100%)	8(88.9%)	37 (88.1%)	33 (82.5%)	2.134	0.545
	Female	13 (13%)	0(0%)	1 (11.1%)	5 (11.9%)	7 (17.5%)		
Pulse rate (beats/minute)	Mean ± SD	100.85±20.46	101.6±13.35	102.7±19.32	103.4±22.54	98±20.46	0.693	0.282
	Median (range)	101.50 (65-110)	102 (88-110)	104.50 (70-110)	104 (65-113)	99 (67-115)		
Respiratory rate (breaths/minute)	Mean ± SD	24.01±5.63	23.6±3.64	28.6±9.24	24.5±5.87	22.6±4.07	3.154	0.153
	Median (range)	23 (15-51)	23 (18-31)	28 (20-51)	24 (17-40)	22 (15-38)		
SBP (mmHg)	Mean ± SD	129±12.6	130.8±15.87	130±19.4	126±17.9	124±11.7	0.557	0.808
	Median (range)	130 (80-150)	140 (80-150)	140 (110-160)	130 (90-160)	130 (90-160)		
DBP (mmHg)	Mean ± SD	79.70±11.59	80.0±11.18	78.9±9.28	82.6±14.98	79.8±8.91	0.692	0.732
	Median (range)	80 (70-90)	80 (50-130)	80 (50-90)	80 (60-90)	80 (60-130)		
Temperature (°F)	Mean ± SD	98.51±1.10	98.2±0.41	98.7±1.02	98.6±0.94	98.3±0.97	1.150	0.293
	Median (range)	98.4 (97.825-98.6)	98.40 (96.60-102)	98.20 (97.40-101)	98.50 (97.6-100.90)	98.40 (96.60-102)		

Table 3 represents the association between oxygen saturation levels, which is divided into four groups: Group A (≤87%, n = 9), Group B (88-92%, n = 9), Group C (93-96%, n = 42), and Group D (97-100%, n = 40), with mortality, hypercapnia, and prolonged hospitalization (>seven days). From the analysis, it was observed that mortality was low across all four groups with no statistical significance difference(p = 0.179). However, compared to Group C (n = 15, 35.71%) and Group D (n = 2, 4.44%), hypercapnia was much more frequent in groups with lower oxygen saturation, namely, Group A (n = 5, 55.56%) and Group B (n = 4, 44.44%) (p < 0.001). Additionally, Group A (n = 3, 33.3%) and Group B (n = 1, 11.1%) had longer hospital stays than Group C (n = 3, 4.8%) and Group D (n = 0, 0%), which was found to be statistically significant (p < 0.001).

**Table 3:** The proportions of patients in each group (categorized based on SpO2% on admission) who experience mortality, prolonged hospitalization, and hypercapnia (analyzed using chi-square test) The data have been presented in frequency (%). p < 0.05 is considered a significance level.

Parameters		Oxygen saturation levels during hospital admission				Chi square	p-value
		Group A (≤87%) (n = 9)	Group B (88-92%) (n = 9)	Group C (93-96%) (n = 42)	Group D (97-100%) (n = 40)		
Mortality	n (%)	0 (0)	1 (10)	0 (0)	2 (4.44)	4.162	0.179
Hypercapnia	n (%)	5 (55.56)	4 (44.44)	15 (35.71)	2 (4.44)	16.906	<0.001
Prolonged hospitalization (>seven days)	n (%)	3 (33.3)	1 (11.1)	2 (4.8)	0(0.00)	15.006	<0.001

One of the severe signs of COPD is the consolidation of the lungs, wherein there is inflammatory exudate present in the lung, causing ventilation-perfusion mismatch in the affected lobe/segment of the lung. Consolidation of lungs was present in 19% of patients, and incidence was expectedly low in Group D (Table 4). NIV was required in seven out of nine (77.78%) cases in Group A and least (3/40; 7.5%) in patients of Group D. The median pO2 was within the normal range (pO2: 75-100 mmHg) in Groups B to D but was below normal (pO2: 66.38 ± 24.75 mmHg) in patients of Group A. The oxygen saturation levels were ≥93-96% in 84% of patients. Moreover, the pCO2 of this Group (A) was also high (pCO2: 67.08 ± 27.3 mmHg; normal range: pCO2: 38-42 mmHg); therefore, seven out of nine needed NIV and prolonged hospitalization. Overall, 21% of patients had elevated pCO2 levels.

**Table 4:** Assessment of chronic obstructive pulmonary disease (COPD) on subjects with different oxygen saturation during hospital admission analysis using chi-square test The data have been presented in frequency (%). p < 0.05 is considered a significance level. AVPU, Alert, Verbal, Pain, Unresponsive

Parameters	Oxygen saturation level during hospital admission				Chi square	p-value
	Group A (≤87%) (n = 9)	Group B (88-92%) (n = 9)	Group C (93-96%) (n = 42)	Group D (97-100%) (n = 40)		
No. of patients having consolidation on imaging	2 (22.22%)	2 (22.22%)	9 (21.43 %)	6 (15%)	0.70	0.874
Required non-invasive ventilation	7 (77.78)	5 (55.56%)	13 (30.95%)	3 (7.5%)	16.27	<0.001
No. of patients with left heart failure.	0	0	4 (9.52%)	1 (2.5%)	3.28	0.350
No. of patients who had Cor pulmonale	1 (11.11%)	1 (11.11%)	5 (11.90%)	0	5.02	0.170
Assessment of AVPU scale for consciousness in relation to oxygenation status						
Alert	7 (77.78%)	7 (70%)	32 (76.19%)	36 (90%)	2.91	0.405
Voice	0	0	2, (4.76%)	0	2.82	-
Pain	2 (22.22%)	3 (33.33%)	2 (4.76%)	2 (5%)	-	0.421
Unresponsiveness	0	0	0	0	-	NS

Four groups with varying oxygen saturation values upon admission are shown in Table 5, with their mean and SD for each blood marker: Group A (≤ 87%), Group B (88-92%), Group C (93-96%), and Group D (97-100%). There was no distinct variation in albumin levels across the groups (p = 0.393). Groups A (108.36) and C (134.98) had considerably higher CRP levels than Group D (46.85) (p < 0.001). Group A had a considerably lower partial pressure of oxygen (pO2) (60.48) in comparison to higher saturation groups, which is statistically significant (p = 0.004). Group A had a considerably higher partial pressure of carbon dioxide (pCO2) at 58.41 ± 24.465 compared to Group D at 37.71 ± 11.756 (p = 0.009). White cell count (WCC) and bicarbonate (HCO3) levels did not change significantly across the groups (p = 0.100 and p = 0.447, respectively).

**Table 5:** Laboratory parameters in the four oxygen saturation groups analysis using ANOVA The continuous data have been presented in terms of mean ± SD. p < 0.05 is considered as significance level using ANOVA. CRP, C-reactive protein

Variables	Admission oxygen saturation group	n	Mean	SD	F value	p-value
Albumin	87% or less	9	3.5	0.278	0.69	0.393
	88-92%	9	3.49	0.615		
	93-96%	42	3.59	0.412		
	97-100%	40	3.69	0.398		
CRP	87% or less	9	108.36	91.672	2.69	<0.001
	88-92%	9	77.43	80.71		
	93-96%	42	134.98	78.943		
	97-100%	40	46.85	50.189		
pO2, kPa	87% or less	9	60.48	21.288	1.96	0.004
	88-92%	9	85.28	49.79		
	93-96%	42	98.26	42.44		
	97-100%	40	93.49	30.094		
pCO2, kPa	87% or less	9	58.41	24.465	1.62	0.009
	88-92%	9	64.04	39.198		
	93-96%	42	51.01	28.049		
	97-100%	40	37.71	11.756		
HCO3	87% or less	9	30.07	6.65	0.69	0.100
	88-92%	9	26.97	10.098		
	93-96%	42	26.49	7.328		
	97-100%	40	24.26	5.125		

COPD is commonly associated with left heart failure in clinical practice, as they share an identical pathogenic mechanism. However, the occurrence of left-sided heart failure in our study was very low (5/100). Cor pulmonale is commonly associated with COPD, but in our study, it occurred in only seven patients.

All the patients were assessed on the Alert, Verbal, Pain, Unresponsive (AVPU) Scale. Around 90% (>92%) of patients with good oxygen saturation status were fully awake (Group C: 88.89%; Group D: 95.56%), and 98 of the 100 responded to the verbal stimulus, except for two patients of the Group C. Most patients of all four groups also responded to pain stimuli.

Although all the patients' mean serum bicarbonate was within the normal range (22-32 mmol/L), 12 patients (Group A: four; Group B: three; Group C: three; Group D: two) had elevated bicarbonate levels, mostly (7/12) in Groups A and B. Low albumin levels (normal range: 3.5-5.5 g/dL) also reflect acute infection; of our patients, 24 had low albumin levels. CRP serum levels are raised in COPD patients due to systemic inflammation. In our study cohort, the CRP levels of all patients in the four groups were markedly high (normal range: 8-10 mg/dL); in Group D, the levels were relatively low. COPD patients with a high baseline WBC have a high probability of long-term acute exacerbation of COPD. In our study cohort, 39 patients had WBC >11,000.

Table 6 provides the clinical outcomes and conditions between Group A+B and Group C+D, based on oxygen saturation level. Mortality was found to be higher (5.26%) than others (2.4%). The difference was not statistically significant (p = 0.179). Group A+B experienced hypercapnia at a considerably higher rate (n = 9, 50.0%) compared to Group C+D (n = 17, 20.7%) (p = 0.010). Similarly, Duration of hospital stay shows a significant difference among the group (p < 0.05).

**Table 6:** Analysis of the impact of initial oxygenation levels on critical parameters using chi-square test The data have been presented in frequency (%). p < 0.05 is considered a significance level.

Parameters	Oxygenation status at admission			
	Group A+B (n = 18)	Group C+D (n = 82)	Chi square	p-value
	SpO2 ≤93-96%	SpO2 ≥ 93-96%		
	% of n	n		
Mortality	1 (5.55)	2 (2.43)	1.81	0.179
Presence of hypercapnia	9 (50.0)	17 (20.7)	6.63	0.010
Duration of hospital stay > seven days	3 (16.67)	3 (3.65)	3.84	<0.05
Left-side heart failure	0 (0.0)	12 (14.6)	2.99	0.084
Cerebrovascular disease	2 (11.1)	5 (6.1)	0.57	0.450
Presence car pulmonale	2 (11.1)	5 (6.1)	1.06	0.807
Alert	13 (72.22)	76 (92.68)	3.84	<0.05
Pain	5 (27.77)	14 (17.07)	4.82	<0.05

Correlation between initial oxygenation status with DECAF scores and NEWS2 scores in COPD patients

Spearman’s rank-order correlations were run to examine the relationship between initial oxygenation status with DECAF scores and NEWS2 scores in COPD patients. There were negative and significant correlations between initial oxygenation status and DECAF scores, rs = -0.28, n = 100, p = 0.004, and initial oxygenation status and NEWS2 scores, rs = -0.49, n = 100, p < 0.001.

The correlation coefficients of -0.28 (between initial oxygenation status and DECAF score) and -0.49 (between initial oxygenation status and NEWS2) show a low negative correlation. The p-value shows significance due to the sample size or the strong association between initial oxygenation status with DECAF and NEWS2, but there are other important determinants as well.

**Figure 2:**
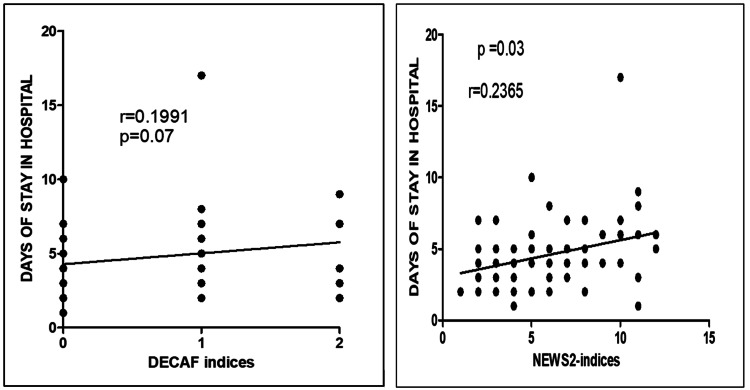
The correlation between the duration of hospital stays and DECAF and NEWS2 score DECAF, Dyspnea, Eosinopenia, Consolidation, Acidemia, and Atrial Fibrillation; NEWS2, National Early Warning Score 2

We found that the DECAF score correlated significantly with pO2 and pCO2, whereas NEWS2 correlated significantly with pCO2, albumin levels, and WBC counts (Table 7). We also found a significant positive relation between the duration of hospital stay with the DECAF and NEWS2 score (Figure 2).

**Table 7:** Correlation of clinical parameters with the DECAF and NEWS2 scores using correlation coefficient DECAF, Dyspnea, Eosinopenia, Consolidation, Acidemia, and Atrial Fibrillation; NEWS2, National Early Warning Score 2

Clinical parameters	DECAF score		NEWS2 score	
	r	p-value	r	p-value
pO2, (mmHg)	0.224	0.0456	0.006	0.9572
pCO2, (mmHg)	0.4117	0.0001	0.3939	<0.001
HCO3, (mmol/L)	0.1355	0.2249	0.5557	0.1624
Albumin(g/dL)	-0.0405	0.047	-0.227	0.0403
WBC counts	-0.0330	0.7685	0.2329	0.0352

## Discussion

A variety of parameters are available for the diagnosis and efficient management of patients suffering from COPD, with the goal of safe and early hospital discharge. Clinicians' foremost concern is to isolate patients at a high risk of dying so that an appropriate level of care is imparted. Therefore, clinicians need support and guidance from well-validated protocols for better patient evaluation. At the same time, especially in low-income settings like India, it becomes important to make them conveniently accessible, affordable, and manageable. This is perhaps a maiden study from India where the impact of the initial oxygen levels on critical determinants of COPD was evaluated. 

The median age of our study cohort was 69 years, and it has been well-established that accelerated aging is associated with the development of COPD [36,37]. Monitoring respiratory rate, breath rate, blood pressure, and body temperature are crucial parameters that aid in treating and managing COPD [38-40]. Our results showed that these parameters were within normal ranges across all oxygen saturation groups, suggesting that initial oxygenation status may not significantly impact these baseline measurements. This finding supports previous research that emphasizes the importance of these vital signs in the management of COPD [39,40].

Our study had a main finding of hypercapnia, especially among patients who had lower SpO2 levels, where the pCO2 and HCO3- levels in these individuals were higher. In previous studies, there have been strong associations between low oxygen saturation and increased incidence of hypercapnia [41-43]. Patients with SpO2 ≤ 87% had twice the chance of experiencing hypercapnia compared to those whose oxygenation levels were higher [44].

Besides higher incidences of hypercapnia, in our study, patients with initial oxygen less than 93-96% also had higher incidences of consolidation, a severe sign of COPD, wherein lungs are filled with inflammatory exudate, further obstructing the breathing of the patients. The incidence of consolidation was 19% and was expectedly low in Group D, with higher oxygenation levels, albeit insignificant. Our data corroborate with a European COPD audit report, where the prevalence of consolidation for hospitalized exacerbations was 19% [45]. The 2014 UK National COPD audit shows that consolidation is associated with increased mortality [46]. However, due to the low mortality in our study, we could not associate the mortality rate with the presence of chest consolidation.

Left-sided heart failure is a common comorbidity in COPD but was found in only a small minority of our patients (5%). There is evidence in the literature that low oxygen saturation and the oxygen desaturation index (ODI) are good correlates for the presence of left-sided heart failure [47,48]. Because this outcome was observed in a small number of patients in the study cohort, such monitoring could be of importance with respect to cardiac functioning in COPD patients with low oxygen saturation status.

NIV is an evidence-based first-line intervention for COPD patients with respiratory distress. It facilitates gas exchange, reduces breathing work, and curtails hospital stay and mortality. Compared to invasive ventilation, NIV causes fewer problems [49]. A Dutch study noted a better survival rate in patients who received NIV when indicated than those who did not receive it due to some valid reasons [50]. In this study, most patients with an initial oxygen level of less than 93-96% needed NIV, and these patients showed an improved sense of well-being with a 3% mortality rate. NIV emerged as an independent factor associated with a significantly lower risk of death [51]. Chawla et al. [52] from India have proposed guidelines for using NIV in acute respiratory failure patients in ICU. Perhaps to make COPD treatment economically viable in low-income countries, an Indian study observed that an early withdrawal did not increase the risk of weaning failure from the NIV [53]. In contrast, prolonged administration of NIV leads to poor outcomes [54].

The DECAF score predicts in-hospital mortality and disease severity and facilitates early discharge of COPD patients [55]. The score integrates typically available indicators at admission. It helps healthcare providers to stratify the patient into risk categories - low risk (score = 0-1), moderate risk (score = 2), and high risk (score = 3-6) - thereby reducing mortality and morbidity by the initiation of the appropriate level of care, besides reducing the cost of the treatment [56]. NEWS2 is primarily used to identify clinically deteriorating patients and is most useful in patients with COPD. In our study, DECAF and NEWS scores correlated well with pCO2 and albumin levels. DECAF score correlated well with pO2, whereas NEWS2 correlated with WBC counts. Moreover, both scores showed a linear relation with the duration of hospital stay, albeit significant with the NEWS2 score only. As there were only three deaths, we could not compare mortality with both scores. 

In an Indian study, the DECAF score was significantly higher (p < 0.001) in patients who died during the study period than those who survived [33]. The DECAF score efficiently predicts inpatient mortality and readmission in COPD management in the UAE study [57].

The limitations of our study include its retrospective design and the lesser number of deaths, which constrained our ability to fully assess the impact of initial oxygenation levels on mortality. Additionally, all patients had DECAF indices ≥ 2, limiting the analysis of COPD complications and their association with mortality. Despite these limitations, our study highlights the importance of initial oxygenation status and risk scores in COPD management, offering valuable insights for clinical practice.

Our study emphasizes the critical role of initial oxygen saturation in predicting clinical outcomes in COPD patients. The integration of DECAF and NEWS2 scores with oxygenation status can enhance patient stratification and optimize treatment strategies, particularly in settings with limited resources. Further research is needed to validate these findings and explore their broader application in diverse clinical contexts.

## Conclusions

This study underscores the critical importance of initial oxygenation status in the management of COPD patients, particularly in the context of resource-limited settings. Our findings demonstrate that patients with lower SpO2 levels (≤93-96%) at admission are significantly more likely to experience adverse outcomes, including hypercapnia, prolonged hospital stays, and an increased need for NIV. The significant correlations between initial oxygenation status and both DECAF and NEWS2 scores further highlight the utility of these indices in predicting patient outcomes. Specifically, the DECAF score's correlation with pO2 and pCO2 and the NEWS2 score's correlation with pCO2, albumin, and WBC counts reinforce their roles as valuable tools in assessing disease severity and guiding treatment decisions. The low mortality observed in this cohort can be attributed to the high baseline SpO2 in most patients. Therefore, our study suggests that early and accurate assessment of oxygenation status, combined with applying DECAF and NEWS2 scores, can improve clinical outcomes in COPD management. These findings advocate for the routine use of these parameters in scores-DECAF and NEWS2 in clinical practice, particularly in settings where cost-effective and rapid decision-making is essential.
